# Social network, perceived satisfaction with neighborhoods and depressive symptoms among older adults in Korea

**DOI:** 10.1038/s41598-025-26332-2

**Published:** 2025-11-27

**Authors:** Jeehye Lee, Ji-Eun Lee, Seonji Kim, Hye Sook Min, Kyung-Shin Lee

**Affiliations:** 1 Department of Preventive Medicine, College of Medicine, Konkuk University, Chungju-si, 27478 South Korea; 2 Department of Human Systems Medicine, College of Medicine, Seoul National University, Seoul, 03080 South Korea; 3 Department of Biomedical Systems Informatics, Yonsei University College of Medicine, Seoul, 03722 South Korea; 4 Yonsei Institute for Digital Health, Yonsei University, Seoul, 03722 South Korea; 5 Public Healthcare Research Institute, National Medical Center, 04564 Seoul, South Korea; 6 Center for Public Healthcare Policy, National Medical Center, 245, Eulji-ro, Jung-gu, Seoul, 04564 South Korea

**Keywords:** Social networks, Perceived satisfaction with the neighborhood, Depressive symptoms, COVID-19 pandemic, Difference-in-differences, Psychology, Environmental social sciences, Risk factors

## Abstract

The coronavirus disease 2019 (COVID-19) pandemic has significantly affected social networks and perceived satisfaction with neighborhoods, potentially affecting the mental health of older adults. This study investigated the changes in these factors and their association with depressive symptoms among Korean older adults before and during the COVID-19 pandemic. The prevalence of depressive symptoms slightly increased during the pandemic (4.7–4.9%). Social networks weakened, whereas perceived neighborhood satisfaction improved. Notably, the protective effect of social networks against depressive symptoms became stronger during the pandemic, particularly among low-income individuals and those living in rural areas. Maintaining social networks is essential for promoting mental health resilience among older adults, especially during crises. Policymakers should thus prioritize interventions that strengthen community cohesion, provide governmental support, and facilitate social networking opportunities for vulnerable populations.

**Supplementary Information:**

The online version contains supplementary material available at 10.1038/s41598-025-26332-2.

## Introduction

Social networks and perceived satisfaction with neighborhoods have a strong effect on the health of an individual, including their mental health^1^. Social isolation, stemming from a lack of social networks, has been shown to increase the risk of heart disease by 29%, stroke by 32% and overall mortality by 26%, even after adjusting for factors that could influence mortality^2^. Social isolation is also closely associated with mental health. According to previous research, social isolation can have affect mental health negatively in the form of impairments like depressive symptoms and cognitive impairment^3^. According to the convoy model of social networks for older adults, neighborhoods are the outermost circle of social relationships^4^. These neighborhoods can be affected by situational characteristics such as neighborhood safety, economic status, and community resources. A prior study has found that safety, transportation, and other factors influence older adults’ social participation and well-being^5^. Healthcare services also affect the health and wellbeing of older adults and interestingly, residential environments and outdoor spaces affect individuals’ social activities and well-being^6^. Through various pathways, these factors contribute to the impact of neighborhoods on older adults’ mental health^7^.

Older people are at a higher risk of depressive symptoms than younger ones^8,9^. The coronavirus disease 2019 (COVID-19) pandemic has further exacerbated mental health vulnerabilities among the older population due to fear and anxiety about infection and a higher mortality rate among older adults^9^. Although there has been no large-scale lockdown, closure of community centers and sports facilities as part of the social distancing guidelines limited social networking opportunities during the pandemic^10^. While some studies suggest that stay-at-home measures, including telecommuting, have improved family relationships, it has concurrently heightened social isolation through the closure of community facilities, sports venues, and senior facilities owing to lockdowns^11,12^. During the COVID-19 pandemic, with the implementation of social distancing measures, elements such as the pandemic itself and the resulting lockdown apparently influenced social networks and the perceived satisfaction of people with their neighborhoods. While the impact of social isolation on mental health is well established, research on the specific relationship between increasing social isolation factors during the pandemic and mental health vulnerability of older adults is still lacking. This study aimed to investigate the changes in social networks and perceived neighborhood satisfaction among the older population before and during the COVID-19 pandemic. Additionally, it also analyzed how the associations between perceived neighborhood satisfaction, social networks, and depressive symptoms evolved before and during the COVID-19 pandemic. In December 2024, Korea entered a super-aged society with an elderly population ratio exceeding 20%^13^, and Korean society is aging rapidly. In this context, awareness and solutions are needed for the potential problems that may arise as the elderly population increases. In particular, the prevalence of depressive symptoms among Korean elderly was reported to be 11.3% as of 2023^13^. This research can provide insights into social isolation and mental health, to which the elderly population is more vulnerable.

## Methods

### Data source and participants

This study used data from the Korea Community Health Survey (KCHS)^14^, conducted in 2019 and 2021. The KCHS, conducted and managed by the Korea Disease Control and Prevention Agency, is a nationally representative survey of Korean adults that collects data on a wide range of health-related topics. The KCHS data were collected through household visits by trained personnel using a laptop-based program called computer-assisted personal interviews (CAPI). The target population of our study was individuals aged over 65 years. The study design and data analysis protocol were reviewed and approved by the Institutional Review Board of the National Medical Center (NMC-2023-03-037). Consent to participate was not applicable for this study because the KDCA hosts raw KCHS data on a public website.

### Variables

Depressive symptoms were the primary outcome variables and were assessed using the Patient Health Questionnaire-9 (PHQ-9), a well-established screening tool for measuring depressive symptoms in individuals^15^. The PHQ-9 is a nine-item self-report questionnaire used to screen for major depressive disorder (MDD). However, the PHQ-9 is also widely used in epidemiological research to assess the severity of depressive symptoms in populations^15,16^. Each item is scored from 0 to 3, producing a total score ranging from 0 to 27, with higher scores indicating greater symptom severity. Given that the PHQ-9 is a symptom-based screening tool rather than a diagnostic instrument, the outcome in this study is referred to as “depressive symptoms” rather than “depressive disorder.” This terminology is consistent with its application in population-based studies to capture depressive symptomatology. Participants with a total score of 10 or higher were classified as having clinically relevant depressive symptoms, in accordance with the KCHS user guidelines and prior validation studies. This threshold has demonstrated high sensitivity (88%) and specificity (88%) for detecting major depression in primary care settings^15^. The PHQ-9 score was treated as a binary variable (≥ 10 vs. <10) to identify elevated depressive symptoms, in line with previous Korean studies using KCHS data^17,18^ and meta-analytic evidence supporting a score of 10 as the optimal clinical cut-off^19^.

The independent variables of interest in this study were two types of perceived satisfaction with neighborhoods, including social network, derived using two questions, and physical environment derived using five questions. The indicators of social networks were assessed based on trust and support from neighbors, and the physical environment was evaluated as being satisfied with neighborhood safety, living environment, and public transportation. Each item was measured using a binary response format (Yes/No), and the full list of questionnaire items is provided in Supplementary Table S1. These items were derived from the Korea Community Health Survey and have been utilized in previous studies investigating neighborhood contexts and mental health among older adults in Korea^20–22^.

To account for potential confounding factors, we controlled for various participant characteristics including sociodemographic (sex, age group, marital status, and education level) and socioeconomic factors (region of residence, occupational status, and monthly income level). Additionally, we controlled for the presence of hypertension and diabetes mellitus, which are potentially confounding health conditions in older adults. The age group was divided into three categories: 65–74, 75–84, and 85 years and over. Educational level was classified as either low (below high school graduation) or high (high school graduation or above). The region of residence (urbanity) was divided into two categories: urban for participants residing in nine major cities and rural for participants residing in nine provinces. Finally, we divided the participants into two groups based on their experiences during the COVID-19 period: “the pre-pandemic group” (2019) and “COVID-19 pandemic group” (2021).

### Statistical analyses

Owing to the complex sampling design of the KCHS, all analyses were conducted using appropriate sampling weights and statistical procedures. We employed the sampling weights provided by the KCHS, which incorporated adjustments for household and individual characteristics, sampling rates, and response rates. Additionally, weights were calibrated to match the sex and age distributions of the registered population in each surveyed area. Further details regarding the sampling weights are available elsewhere^14^.

Weighted percentages generated using the PROC SURVEYFREQ were employed to compare the general characteristics of the participants across regional and household composition categories. These weighted percentages provided population-level estimates for each category. We compared the general characteristics of the participants in the depression group during the COVID-19 pandemic to those before the pandemic and evaluated the significance of the change between these two periods using the Rao-Scott chi-square test. To assess the association between perceived neighborhood satisfaction and depression among the older population, odds ratios (ORs) and 95% confidence intervals (CIs) were calculated using binary multivariable logistic regression analysis (PROC SURVEYLOGISTIC) after adjusting for potential confounding variables over the two periods. To explore the relationships between social network indicators, perceived neighborhood satisfaction, and associated depressive symptoms, we employed a multivariate difference-in-differences (DID) regression approach^23^. The DID analysis is a powerful tool for minimizing the impact of unobserved, time-invariant factors on the analysis of temporal trends by comparing the results before and during policy reform^24^. This analysis allowed us to compare the impact of social networks and the perceived satisfaction with neighborhoods before and during the COVID-19 pandemic on depressive symptoms on older adults. To identify the impact of COVID-19 on regional and socioeconomic differences in these associations, we stratified them based on the urbanity and income levels of the model before and during the pandemic. In addition, to verify the parallel trend assumption in our study, we examined the trend of the association between social networks and the perceived satisfaction and depressive symptoms by socioeconomic level over three years (2017, 2019, and 2021). All statistical analyses were performed using Statistical Analysis System (SAS) software (version 9.4; SAS Institute, Cary, NC, USA). Statistical significance was set at *p* < 0.05.

## Results

Table 1 presents the basic characteristics of the participants and the prevalence of depressive symptoms before and during the COVID-19 pandemic. It was noted that the prevalence of depressive symptoms increased from 4.7% before COVID-19 to 4.9% during the pandemic. Among individuals aged 65–74 years, the prevalence of depressive symptoms was 3.2% before COVID-19 and 3.4% during the pandemic, with a reported increase with the progression of age, reaching about three times higher among those aged 85 years and above. The prevalence of depressive symptoms among males was 3.2% before the COVID-19 outbreak and 3.3% during the pandemic, whereas it among females was 5.9% before the COVID-19 outbreak and 6.2% during the pandemic. It was further noted that individuals with higher education had a lower depressive symptom rate (2.9%) than those with lower education (5.6%). The prevalence of depressive symptoms was 5.2% in the higher income group and 3.0% in the lower income group, indicating minimal changes during the pandemic. Among those without an occupation, the prevalence of depressive symptoms increased from 5.9 to 6.3%, whereas among those with an occupation, it decreased from 2.0 to 1.9%. The prevalence of depressive symptoms among individuals who were single, divorced, or widowed increased from 7.1% before the COVID-19 pandemic to 7.6% during the pandemic, whereas that among those who lived with a partner remained similar before and during the pandemic, at 3.5% and 3.4%, respectively.

**Table 1:** Basic characteristics of the participants.

Variables	Category	Before the COVID-19 pandemic			During the COVID-19 pandemic		
		Total	Depression (Yes)	Depression (No)	Total	Depression (Yes)	Depression (No)
		*N*(%)	*N*(%)	*N*(%)	*N*(%)	*N*(%)	*N*(%)
Total		69,124(100.0)	3164(4.7)	65,960(95.3)	71,317(100.0)	3208(4.9)	68,109(95.1)
Age group	65–74	36,159(57.0)	1121(3.2)	35,038(96.8)	38,409(58.3)	1169(3.4)	37,240(96.6)
	75–84	27,494(36.5)	1536(6.2)	25,958(93.8)	26,442(34.0)	1450(6.3)	24,992(93.7)
	85+	5471(6.6)	507(9.9)	4964(90.1)	6466(7.7)	589(10.2)	5877(89.8)
Sex	Male	28,635(44.4)	825(3.2)	27,810(96.8)	30,005(44.8)	871(3.3)	29,134(96.7)
	Female	40,489(55.6)	2339(5.9)	38,150(94.1)	41,312(55.2)	2337(6.2)	38,975(93.8)
Education	Below High school graduation	53,713(68.1)	2769(5.6)	50,944(94.4)	52,387(63.1)	2725(6.1)	49,662(93.9)
	High school graduation and above	15,411(31.9)	395(2.9)	15,016(97.1)	18,930(36.9)	483(2.9)	18,447(97.1)
Monthly income	Under 4 million won	54,626(78.1)	2756(5.2)	51,870(94.8)	54,244(75.9)	2681(5.5)	51,563(94.5)
	4 million won and more	14,498(21.9)	408(3.0)	14,090(97.0)	17,073(24.1)	527(3.1)	16,546(96.9)
Occupation	Yes	27,654(30.2)	606(2.0)	27,048(98.0)	29,305(31.4)	599(1.9)	28,706(98.1)
	No	41,470(69.8)	2558(5.9)	38,912(94.1)	42,012(68.6)	2609(6.3)	39,403(93.7)
Marital status	Live together (Married)	44,055(66.3)	1460(3.5)	42,595(96.5)	44,574(64.7)	1429(3.4)	43,145(96.6)
	Separated (Single or Divorced/Widowed)	25,069(33.7)	1704(7.1)	23,365(92.9)	26,743(35.3)	1779(7.6)	24,964(92.4)
The presence of hypertension or diabetes mellitus	Yes	42,515(61.4)	2108(5.2)	40,407(94.8)	44,335(61.0)	2199(5.5)	42,136(94.5)
	No	26,609(38.6)	1056(4.1)	25,553(95.9)	26,982(39.0)	1009(4.0)	25,973(96.0)
Urbanity	Rural	52,813(57.7)	2393(4.7)	50,420(95.3)	53,850(56.9)	2359(4.9)	51,491(95.1)
	Urban	16,311(42.3)	771(4.7)	15,540(95.3)	17,467(43.1)	849(4.9)	16,618(95.1)

No. of variables is unweighted and percentage (%) is weighted.

Table 2 describes how social networks and older adults’ perceived satisfaction with their neighborhoods changed before and during the COVID-19 pandemic. All variables related to social networks and perceived satisfaction with neighborhoods showed statistically significant changes before and during the pandemic. The exchange of help with neighbors decreased from 55.3 to 51.2%, whereas the percentages observing the perceived satisfaction of older adults with the neighborhood variables increased during the COVID-19 pandemic. The prevalence of depressive symptoms among individuals who responded ‘no’ to “Trust on neighbors” increased from 6.4 to 7.8%. The prevalence of depressive symptoms decreased from 4.1 to 3.5% among those who answered ‘yes’ to exchanging help with neighbors. For those responding ‘no’ to the respective questions, the prevalence of depressive symptoms increased from 5.5 to 6.3% for “Exchange help with neighbors” and from 7.9 to 9.0% for “Perceived satisfaction with overall safety.”

**Table 2:** Descriptive statistics of the indicators of perception of neighborhood during the COVID-19 pandemic compared to before the pandemic.

Indicators	Category	Before the COVID-19 pandemic (*N* = 69114)			During the COVID-19 pandemic (*N* = 71311)			Rao Scott χ2 or t (*P* value)
		Total	Depression (Yes)	Depression (No)	Total	Depression (Yes)	Depression (No)	
		N(%)	N(%)	N(%)	N(%)	N(%)	N(%)	
Trust of neighbors	Yes	56,453(74.4)	2303(4.1)	54,150(95.9)	57,375(73.4)	2172(3.9)	55,203(96.1)	5.95(0.015)
	No	12,671(25.6)	861(6.4)	11,810(93.6)	13,942(26.6)	1036(7.8)	12,906(92.2)	
Exchanging help with neighbors	Yes	50,585(55.3)	2033(4.1)	48,552(95.9)	49,665(51.2)	1835(3.6)	47,830(96.4)	67.76(< 0.0001)
	No	18,539(44.7)	1131(5.5)	17,408(94.5)	21,652(48.8)	1373(6.3)	20,279(93.7)	
Perceived satisfaction with overall safety	Yes	63,248(88.7)	2691(4.3)	60,557(95.7)	66,283(90.7)	2763(4.5)	63,520(95.5)	49.53(< 0.0001)
	No	5876(11.3)	473(7.9)	5403(92.1)	5034(9.3)	445(9.0)	4589(91)	
Perceived satisfaction with natural	Yes	61,166(85.5)	2684(4.4)	58,482(95.6)	64,611(88.1)	2796(4.7)	61,815(95.3)	62.99(< 0.0001)
	No	7958(14.5)	480(6.4)	7478(93.6)	6706(11.9)	412(6.8)	6294(93.2)	
Perceived satisfaction with living	Yes	62,073(89.4)	2697(4.4)	59,376(95.6)	65,551(91.6)	2818(4.6)	62,733(95.4)	74.30(< 0.0001)
	No	7051(10.6)	467(7.3)	6584(92.7)	5766(8.4)	390(8.0)	5376(92)	
Perceived satisfaction with traffic	Yes	52,888(80.6)	2144(4.2)	50,744(95.8)	55,389(81.8)	2229(4.4)	53,160(95.6)	9.61(0.002)
	No	16,236(19.4)	1020(6.7)	15,216(93.3)	15,928(18.2)	979(7.2)	14,949(92.8)	
Perceived satisfaction with health services access	Yes	53,962(81.8)	2178(4.2)	51,784(95.8)	56,582(83.9)	2294(4.5)	54,288(95.5)	31.75(< 0.0001)
	No	15,162(18.2)	986(6.9)	14,176(93.1)	14,735(16.1)	914(7.1)	13,821(92.9)	

No. of variables is unweighted and percentage (%) is weighted.

Table 3 presents the adjusted odds ratios for depressive symptoms based on social networks and perceived satisfaction with neighborhoods before and during COVID-19 pandemics. This table also includes the values obtained from the DID analysis. All variables had a significant impact on depressive symptoms, with odds ratios consistently of < 1. The DID analysis indicated that variables related to social networks had statistically significant positive values (Wald χ^2^ for trust of neighbors was 7.29 [*P* value: 0.007], and Wald χ^2^ for exchange help with neighbors was 4.81 [*P* value: 0.028], respectively).

**Table 3:** Association between indicators of perception of neighborhood and depressive symptoms before and during the COVID-19 Pandemic.

Indicators	Depressive symptom		Difference-in differences
	Before the COVID-19 pandemic	During the COVID-19 pandemic	Wald χ2 (*P* value)
	aOR (95% CI)	aOR (95% CI)	
Trust of neighbors (Yes)	0.62(0.56, 0.69)	0.48(0.44, 0.53)	7.29(0.007)
Exchanging help with neighbors (Yes)	0.73(0.66, 0.81)	0.57(0.51, 0.62)	4.81(0.028)
Perceived satisfaction with overall safety (Yes)	0.53(0.47, 0.61)	0.49(0.43, 0.56)	2.58(0.108)
Perceived satisfaction with natural (Yes)	0.63(0.56, 0.72)	0.63(0.55, 0.72)	0.24(0.624)
Perceived satisfaction with living (Yes)	0.57(0.50, 0.65)	0.54(0.47, 0.62)	0.72(0.398)
Perceived satisfaction with traffic (Yes)	0.63(0.57, 0.70)	0.60(0.54, 0.67)	0.09(0.770)
Perceived satisfaction with health services access (Yes)	0.60(0.54, 0.67)	0.62(0.56, 0.70)	0.84(0.360)

The model was adjusted for age group, sex, education, monthly income, occupation, marital status, the presence of hypertension or diabetes mellitus, and urbanity.

Table 4 divided participants into low-income and high-income groups and presented adjusted odds ratios and DID for depression based on social networks and the perceived satisfaction with neighborhoods before and during COVID-19. In the low-income group, odds ratios for depression ranged from 0.54 to 0.68 when participants answered ‘yes’ to all variables. This trend remained the same during the COVID-19 pandemic. The DID analysis showed statistically significant high values for variables related to social networks (Wald χ^2^ for trust of neighbors was 4.41 [*P* value: 0.036]), Wald χ^2^ for exchange help with neighbors was 6.15 [*P* value: 0.013], and Wald χ^2^ for perceived satisfaction with overall safety was 3.96 [*P* value: 0.047]), respectively. Among the high-income group, the odds ratio of depression for the groups who answered “yes” to the question regarding “Trust on neighbors”, and “Exchanging help with neighbors” were 1.12 and 1.07, which were not statistically significant. However, during the COVID-19 pandemic, the adjusted odds ratios for depression decreased in the group that answered ‘yes’ to social network variables (trust of neighbors: aORs was 0.40 [95% CI: 0.32, 0.49], and “Exchanging help with neighbors” 0.55 [95% CI: 0.50, 0.62]). The DID value was statistically significant for the “Trust of neighbors” variable at 6.15 (*P* value: 0.013).

**Table 4:** Association between indicators of perception of neighborhood and depressive symptoms before and during the COVID-19 pandemic according to monthly income.

Indicators	Monthly income (low)		DID	Monthly income (high)		DID
	Before the COVID-19 pandemic	During the COVID-19 pandemic	Wald χ2 (*P* value)	Before the COVID-19 pandemic	During the COVID-19 pandemic	Wald χ2 (*P* value)
	aOR(95% CI)	aOR(95% CI)		aOR(95% CI)	aOR(95% CI)	
Trust of neighbors (Yes)	**0.57(0.51**,** 0.64)**	**0.50(0.45**,** 0.56)**	**4.41(0.036)**	**1.12(0.88**,** 1.42)**	**0.40(0.32**,** 0.49)**	**6.15(0.013)**
Exchanging help with neighbors (Yes)	**0.68(0.61**,** 0.76)**	**0.55(0.50**,** 0.62)**	**6.15(0.013)**	1.07(0.84, 1.37)	0.63(0.52, 0.76)	0.13(0.718)
Perceived satisfaction with overall safety (Yes)	**0.54(0.47**,** 0.62)**	**0.50(0.44**,** 0.57)**	**3.96(0.047)**	0.50(0.37, 0.69)	0.46(0.35, 0.60)	NA
Perceived satisfaction with natural (Yes)	0.65(0.57, 0.75)	0.64(0.55, 0.74)	0.87(0.352)	0.54(0.42, 0.69)	0.60(0.45, 0.79)	1.33(0.248)
Perceived satisfaction with living (Yes)	0.60(0.52, 0.69)	0.55(0.48, 0.64)	1.09(0.296)	0.44(0.33, 0.58)	0.46(0.33, 0.62)	NA
Perceived satisfaction with traffic (Yes)	0.63(0.57, 0.70)	0.62(0.56, 0.70)	0.19(0.663)	0.63(0.49, 0.79)	0.50(0.40, 0.62)	0.06(0.802)
Perceived satisfaction with health services access (Yes)	0.61(0.54, 0.68)	0.64(0.57, 0.72)	0.33(0.567)	0.58(0.47, 0.73)	0.55(0.45, 0.69)	1.16(0.281)

The model was adjusted for age group, sex, education, occupation, marital status, the presence of hypertension or diabetes mellitus, and urbanity; “NA” presented “not available” due to lack of sample size.

Statistically significant DID estimates are shown in bold.

Table 5 presents the results of the same analysis when the participants were divided into urban and rural categories. The DID analysis indicated a statistically significant positive value in the “Trust of neighbors” variable for the group that answered ‘yes’ during the COVID-19 pandemic.

**Table 5:** Association between indicators of perception of neighborhood and depressive symptoms before and during the COVID-19 pandemic according to urbanity.

Indicators	Rural		DID	Urban		DID
	Before the COVID-19 pandemic	During the COVID-19 pandemic	Wald χ2 (*P* value)	Before the COVID-19 pandemic	During the COVID-19 pandemic	Wald χ2 (*P* value)
	aOR(95% CI)	aOR(95% CI)		aOR(95% CI)	aOR(95% CI)	
Trust of neighbors (Yes)	**0.60(0.53, 0.69)**	**0.47(0.42, 0.54)**	**6.06(0.014)**	0.63(0.54, 0.75)	0.49(0.42, 0.57)	0.78(0.376)
Exchanging help with neighbors (Yes)	0.74(0.66, 0.84)	0.58(0.51, 0.66)	2.15(0.143)	0.71(0.59, 0.85)	0.54(0.46, 0.64)	1.81(0.179)
Perceived satisfaction with overall safety (Yes)	0.50(0.43, 0.59)	0.41(0.34, 0.48)	NA	0.58(0.47, 0.72)	0.62(0.52, 0.75)	0.00(0.951)
Perceived satisfaction with natural (Yes)	0.59(0.50, 0.69)	0.60(0.50, 0.71)	0.07(0.785)	0.69(0.56, 0.85)	0.67(0.55, 0.82)	0.02(0.878)
Perceived satisfaction with living (Yes)	0.55(0.47, 0.64)	0.49(0.41, 0.58)	0.30(0.586)	0.61(0.47, 0.79)	0.62(0.49, 0.77)	0.42(0.517)
Perceived satisfaction with traffic (Yes)	0.64(0.57, 0.72)	0.66(0.58, 0.75)	0.12(0.726)	0.60(0.49, 0.74)	0.50(0.42, 0.61)	0.10(0.755)
Perceived satisfaction with health services access (Yes)	0.66(0.59, 0.75)	0.65(0.57, 0.73)	0.03(0.856)	0.50(0.40, 0.61)	0.58(0.47, 0.71)	1.75(0.186)

The model was adjusted for age group, sex, education, monthly income, occupation, marital status, and the presence of hypertension or diabetes mellitus;. “NA” presented “not available” due to lack of sample size.

Statistically significant DID estimates are shown in bold.

In Supplementary Figure S1, we test the validation of parallel trends assumption in the DID analysis by examining the yearly changes in exposure to social networks, perceived satisfaction with neighborhoods, and depressive symptoms from 2017 to 2021. In addition, we conducted statistical interaction tests between time and group using pre-intervention data (2017–2019) to formally assess the parallel trends assumption for each independent variable. Six out of the seven perception indicators showed no statistically significant interaction terms (*p* > 0.05), supporting the overall validity of the DID approach. One variable perceived willingness to exchange help with neighbors demonstrated a statistically significant interaction (*p* = 0.039), suggesting a potential deviation from the parallel trend. Therefore, the DID results for this specific variable should be interpreted with caution. The detailed *p*-values for each indicator are presented in Supplementary Table S2.

We conducted an association analysis between social networks or perceived neighborhood satisfaction and depressive symptoms stratified by covariates, including occupation, marital status, and chronic diseases, to test for differences between these groups. We found that social networks had a protective effect on depressive symptoms in the unemployed, separated, and chronic disease groups and that the associations between social networks and depressive symptoms changed significantly before and during the COVID-19 pandemic (Supplementary Table S3, Table S4, and Table S5).

## Discussion

The study findings indicated that social networking decreased, and the perceived satisfaction with neighborhoods increased among older adults during the COVID-19 pandemic. We found that social networks and perceived satisfaction with one’s neighborhood had a protective effect on depressive symptoms. Interestingly, during the pandemic, the protective effect of network variables on depressive symptoms became stronger than perceived satisfaction with neighborhood. The results showed slightly different patterns when divided by household income level.

The social network variable had no protective effect on depression in the high-income group before the COVID-19 pandemic; however, a protective effect appeared during the COVID-19 pandemic. When analyzing urban and rural areas separately during the COVID-19 pandemic, it was observed that the protective effect of social networks continued to exist in urban areas but did not show significant changes in magnitude before and during the pandemic. In contrast, in rural areas, the magnitude of the protective effect of social networks increased significantly during the pandemic period.

The prevalence of depression among older adults increased slightly before the COVID-19 pandemic (from 4.7 to 4.9%). In general, the pandemic and the lockdowns have been known to have adverse effects on mental health^17^. Our results showed a smaller increase in depressive symptoms than the one previously reported. Another study in Korea reported an increase from 2.8% in 2002 to 5.3% in 2013^25^. We confirmed that the prevalence of depressive symptoms increased with age, was higher in females than males, was lower in those with higher education, and was higher in those without an occupation, in those living alone, and in those with chronic diseases. These results are consistent with those of previous studies^12^ in which the trends did not change before and during the pandemic. It has been shown that having more resources in life can help a person cope better with mental health risks in the midst of a crisis^14^.

In this study, we observed an increase in the perceived satisfaction with neighborhoods compared to before the COVID-19 pandemic. This phenomenon may be partly explained by government-led preventive measures, support for medical expenses, distribution of relief funds, and active surveillance. The government conducted active surveillance through regular phone calls to individuals exposed to early infection during the epidemic and provided isolation supplies^26^. Additionally, relief funds were provided to all citizens, full medical expense coverage was provided to those infected with COVID-19, and support was given for ambulance transportation after allocation to medical institutions^16^. According to previous studies, people have reported an increase in trust in society, individuals, and the government^27^. The increased satisfaction with the neighborhood environment, including satisfaction with accessibility to medical institutions and safety, in this study is likely to be in this context.

Social trust and exchanges of help with neighbors have decreased. The decrease in can be predicted to result from reduced physical interactions with neighbors due to social distancing imposed due to the lockdowns and fear of infection. The long-term consequences of social isolation during pandemics should not be overlooked. Social isolation has been associated with a myriad of adverse health outcomes, including increased risk of cardiovascular disease, stroke, dementia, and mortality^19^. While social networks offer protection against depression, they are also vulnerable to disruption during pandemics. Lockdown measures, physical distancing, and fear of infection can lead to reduced social interactions and a decline in the size and strength of social networks. This paradox poses a significant challenge to population health, as the mental health benefits of social networks are diminished at a time when they are needed most. To mitigate the adverse effects of social isolation during pandemics, proactive policy interventions are essential. These interventions should focus on promoting and facilitating non-face-to-face social networking opportunities, especially for vulnerable populations.

The increased protective effect of social networking against depressive symptoms, as demonstrated by Difference-in-Differences (DID) analysis, can be explained from various theoretical perspectives. The “tend-and-befriend” theory provides a particularly useful framework for understanding this phenomenon^28^. According to this theory, in stressful situations, individuals (particularly women) tend to not only exhibit a ‘fight-or-flight’ response, but also engage in nurturing behaviors (tend) to protect themselves and those close to them, and strengthen social bonds (befriend). In the unprecedented stressful circumstances of the COVID-19 pandemic, this “tend-and-befriend” response may have become more pronounced. As individuals experienced threats to their health, economic stability, and daily routines, they likely invested more actively in social connections that provided a sense of safety and security. This behavior may have functioned as an adaptive strategy that went beyond a mere survival mechanism to protect psychological well-being.

Interestingly, although wealthier individuals showed no significant protective effects of social networks before the pandemic, this effect emerged among them during the COVID-19 pandemic. High-income individuals may have previously maintained their mental health through their economic resources and personal capabilities. They had access to various resources such as professional medical services, personal trainers, and individual hobby activities, which may have reduced their reliance on social networks^29^. However, lockdown measures and social distancing due to the pandemic limited access to these formal and commercial resources^30^, potentially increasing the importance of informal social networks^31^. Furthermore, the pandemic was an unprecedented crisis affecting individuals across all income levels^32^. In such circumstances, economic resources alone may have been insufficient to maintain psychological stability, making social connections and support increasingly important. High-income groups also experienced uncertainty, fear, and feelings of loss, and in these situations, social networks likely functioned as crucial coping mechanisms.

Furthermore, the analysis revealed a greater protective effect in individuals living in rural areas than in those in urban areas. This suggests that the close-knit community ties found in rural settings during the pandemic may have amplified the buffering role of social support networks. Additionally, the lower rate of COVID-19 infection and less stringent social distancing measures in rural areas compared with those in urban environments might have contributed to this observed difference. On the other hand, the fact that the protective effect of social networks continued to exist in urban areas but did not show significant changes in magnitude before and during the pandemic, while in rural areas, the magnitude of the protective effect of social networks increased significantly during the pandemic period, may be attributed to differences in resource accessibility. Rural areas tend to have more limited access to formal support systems such as healthcare services and mental health support compared to urban areas^33,34^. In such circumstances, informal social networks likely served as more crucial support mechanisms, with their effects becoming particularly pronounced during the pandemic.

This study had some limitations that need to be addressed. The first is the issue of measurement error. The relatively modest increase in the prevalence of depressive symptoms among the older population during the COVID-19 pandemic compared to previous research could be attributed to measurement errors. However, because this study primarily aimed to examine the magnitude of the protective effects of social networks and perceived satisfaction with neighborhoods, this can be considered a minor limitation. Furthermore, the mechanisms underlying each of these factors were not elucidated in this study. Thus, future studies should explore how contextual factors influence change. These limitations should be addressed in future studies. Finally, all measures in this study relied on self-reports, which may have introduced potential biases, including recall bias and particularly measurement bias in the PHQ-9 assessment.

Moreover, due to data limitations, we were unable to include certain psychosocial variables such as individual resilience and duration of residence in the community. These unmeasured factors may influence depressive symptoms and contribute to unobserved heterogeneity. To partially address this concern, we included a broad range of sociodemographic and health-related covariates such as education, income, marital status, and chronic disease which may serve as proxies for psychosocial context. Nonetheless, the possibility of residual confounding remains, and future studies should incorporate direct measures of psychosocial resources to further validate and strengthen the findings.

This study provides valuable insights into the complex interplay among social networks, perceived neighborhood satisfaction, and mental health outcomes during the COVID-19 pandemic among older adults in South Korea. Our findings highlight the importance of social networks and neighborhood perceptions in shaping mental health outcomes, particularly during crises. The observed increase in social trust during the pandemic, along with enhanced protective effects of social networks against depressive symptoms, underscores the importance of maintaining and fostering supportive social connections, particularly among vulnerable populations. Policymakers should consider these findings when designing interventions to promote mental health resilience during pandemics, particularly focusing on the needs of older adults in both urban and rural communities. Initiatives aimed at strengthening community cohesion, providing governmental support, and facilitating social networking opportunities can play a crucial role in mitigating adverse mental health impacts of crises such as the recent COVID-19 pandemic.

## Supplementary Information

Below is the link to the electronic supplementary material.

Supplementary Material 1

## Data Availability

The data of Korea Community Health Survey (KCHS) are publicly available through the Community Health Survey website ( [https://chs.cdc.go.kr](https:/chs.cdc.go.kr) ).

## References

[1] Fiori, K. L., Antonucci, T. C. & Cortina, K. S. Social network typologies and mental health among older adults. *J. Gerontol. Ser. B Psychol. Sci. Soc. Sci.* **61**, P25-32. 10.1093/geronb/61.1.p25 (2006). 10.1093/geronb/61.1.p25 16399938

[2] Steptoe, A., Shankar, A., Demakakos, P. & Wardle, J. Social isolation, loneliness, and all-cause mortality in older men and women. *Proc. Natl. Acad. Sci. U.S.A.* **110**, 5797–5801. 10.1073/pnas.1219686110 (2013). 23530191 10.1073/pnas.1219686110 PMC3625264

[3] Brown, V., Morgan, T. & Fralick, A. J. G. P. Isolation and mental health: Thinking outside the box. 34 (2021). 10.1136/gpsych-2020-100461 PMC8149428 34131627

[4] Fuller, H. R., Ajrouch, K. J. & Antonucci, T. C. J. J. o. F. T. & Review. The convoy model and later-life family relationships. **12**, 126–146 (2020). 10.1111/jftr.12376 PMC7283809 32536976

[5] Adler, G. & Rottunda, S. J. J. o. A. s. Older adults’ perspectives on driving cessation. **20**, 227–235 (2006).

[6] Lehning, A. J., Smith, R. J. & Dunkle, R. E. Age-friendly environments and self-rated health: An exploration of Detroit elders. *Res. Aging* **36**, 72–94. 10.1177/0164027512469214 (2014). 25651601 10.1177/0164027512469214

[7] Wandersman, A. & Nation, M. Urban neighborhoods and mental health. Psychological contributions to Understanding toxicity, resilience, and interventions. *Am. Psychol.* **53**, 647–656 (1998). 9633265

[8] Wilkinson, P., Ruane, C. & Tempest, K. Depression in older adults. BMJ **363**, k4922. 10.1136/bmj.k4922 (2018). 10.1136/bmj.k4922 30487197

[9] Zenebe, Y., Akele, B., Selassie, W., Necho, M. & M. & Prevalence and determinants of depression among old age: A systematic review and meta-analysis. *Ann. Gen. Psychiatry* **20**, 55. 10.1186/s12991-021-00375-x (2021). 34922595 10.1186/s12991-021-00375-x PMC8684627

[10] Lee, E. J. & Kim, S. J. Prevalence and related factors of depression before and during the COVID-19 pandemic: Findings from the Korea National health and nutrition examination survey. *J. Korean Med. Sci.* **38**, e74. 10.3346/jkms.2023.38.e74 (2023). 36918028 10.3346/jkms.2023.38.e74 PMC10010913

[11] Partington, L. C., Mashash, M. & Hastings, P. D. Family thriving during COVID-19 and the benefits for children’s Well-Being. *Front. Psychol.* **13** (2022). 10.3389/fpsyg.2022.879195 PMC9135131 35645847

[12] Gayatri, M. & Puspitasari, M. D. The impact of COVID-19 pandemic on family well-being: a literature review. *Fam J. Alex Va.* **5**:10664807221131006. 10.1177/10664807221131006 (2022).

[13] Korea, S. *Elderly Welfare*. https://kosis.kr/search/search.do?query=%EB%85%B8%EC%9D%B8%EB%B3%B5%EC%A7%80 (2025).

[14] Kang, Y. W. et al. Korea community health survey data profiles. *Osong Public Health Res. Perspect.* **6**, 211–217 (2015). 26430619 10.1016/j.phrp.2015.05.003 PMC4551141

[15] Kroenke, K., Spitzer, R. L. & Williams, J. B. The PHQ-9: Validity of a brief depression severity measure. *J. Gen. Intern. Med.* **16**, 606–613 (2001). 11556941 10.1046/j.1525-1497.2001.016009606.x PMC1495268

[16] Sun, Y. et al. The reliability and validity of PHQ-9 in patients with major depressive disorder in psychiatric hospital. *BMC Psychiatry* **20**, 1–7 (2020). 32993604 10.1186/s12888-020-02885-6 PMC7525967

[17] Bae, J. B. et al. The impact of the COVID-19 pandemic on depression in community-dwelling older adults: A prospective cohort study. *Psychol. Med.* **53**, 2992–2999. 10.1017/S0033291721005018 (2023). 37449487 10.1017/S0033291721005018 PMC8692844

[18] Lee, J., Choi, K. S. & Yun, J. A. The effects of sociodemographic factors on help-seeking for depression: Based on the 2017–2020 Korean community health survey. *PloS One* **18**, e0280642 (2023). 36656907 10.1371/journal.pone.0280642 PMC9851551

[19] Manea, L., Gilbody, S. & McMillan, D. Optimal cut-off score for diagnosing depression with the patient health questionnaire (PHQ-9): A meta-analysis. *CMAJ* **184**, E191–E196 (2012). 22184363 10.1503/cmaj.110829 PMC3281183

[20] Lee, J. A., Park, J. H. & Kim, M. Social and physical environments and self-rated health in urban and rural communities in Korea. *Int. J. Environ. Res. Public Health* **12**, 14329–14341 (2015). 26569279 10.3390/ijerph121114329 PMC4661651

[21] Yim, D. H. & Kwon, Y. Does young adults’ neighborhood environment affect their depressive mood? Insights from the 2019 Korean community health survey. *Int. J. Environ. Res. Public Health* **18**, 1269 (2021). 33572580 10.3390/ijerph18031269 PMC7908501

[22] Kim, Y. & Lim, M. K. The potential role of perceived neighborhood social cohesion on COVID-19 vaccination uptake among individuals aged 50 and older: Results from the Korean community health survey. *PloS One* **19**, e0312309 (2024). 39436943 10.1371/journal.pone.0312309 PMC11495590

[23] Lee, M. & Kang, C. Identification for difference in differences with cross-section and panel data. *Econ. Lett.* **92**, 270–276 (2006).

[24] Djernes, J. K. Prevalence and predictors of depression in populations of elderly: A review. *Acta Psychiatr. Scand.* **113**, 372–387. 10.1111/j.1600-0447.2006.00770.x (2006). 10.1111/j.1600-0447.2006.00770.x 16603029

[25] Kim, G. E., Jo, M. W. & Shin, Y. W. Increased prevalence of depression in South Korea from 2002 to 2013. *Sci. Rep.* **10**, 16979 (2020). 33046758 10.1038/s41598-020-74119-4 PMC7550589

[26] Sen, K., Prybutok, G. & Prybutok, V. The use of digital technology for social wellbeing reduces social isolation in older adults: A systematic review. *SSM Popul. Health* **17**, 101020. 10.1016/j.ssmph.2021.101020 (2022). 35024424 10.1016/j.ssmph.2021.101020 PMC8733322

[27] Kye, B. & Hwang, S. J. Social trust in the midst of pandemic crisis: Implications from COVID-19 of South Korea. *Res. Social Strati. Mobil.* **68**, 100523. 10.1016/j.rssm.2020.100523 (2020). 10.1016/j.rssm.2020.100523 PMC7301785 32572302

[28] E Taylor, S. Tend and befriend theory. *J. H O T O S P*. **1**, 32–49 (2012).

[29] Niemeyer, H. & Knaevelsrud, C. Socioeconomic status and access to psychotherapy. *J. Clin. Psychol.* **79**, 937–953. 10.1002/jclp.23449 (2023). 36251952 10.1002/jclp.23449

[30] Koh, D. COVID-19 lockdowns throughout the world. *Occup. Med.* **70**, 322–322. 10.1093/occmed/kqaa073 (2020).

[31] *Why informal networks will be key to the COVID-19 recovery*. https://www.weforum.org/stories/2020/04/covid-19-why-informal-networks-will-be-key/?utm_source=chatgpt.com.

[32] International Labour Organization (ILO). *O. f. E. C.-o. a. D. O. The Impact of the COVID-19 Pandemic on Jobs and Incomes in G20 Economies* (ILO-OECD, 2020).

[33] Lee, J. A., Park, J. H. & Kim, M. Social and physical environments and self-rated health in urban and rural communities in Korea. *Int. J. Environ. Res. Public Health*. **12**, 14329–14341. 10.3390/ijerph121114329 (2015). 10.3390/ijerph121114329 PMC4661651 26569279

[34] Kang, H. & Kim, D. H. Socioeconomic, health, and social connectedness factors associated with self-rated health of octogenarians and nonagenarians in South korea: Urban and rural comparison. *BMC Public Health* **24**, 3477. 10.1186/s12889-024-20984-x (2024). 10.1186/s12889-024-20984-x PMC11658210 39696143

