# Supplementary material for: Social network, perceived satisfaction with neighborhoods and depressive symptoms among older adults in Korea
Source: Sci Rep. 2025 Nov 27;15:42319. doi: 10.1038/s41598-025-26332-2 (PMC12660700; doi:10.1038/s41598-025-26332-2)
Supplement: Supplementary file 1 — Supplementary Material 1 [file 41598_2025_26332_MOESM1_ESM.docx]

**Figure S1. The result of the association between perception of neighborhood and depression from 2017 to 2021 to check the parallel trend**

| 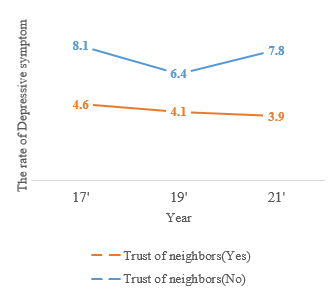 | 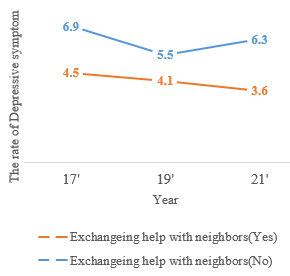 | 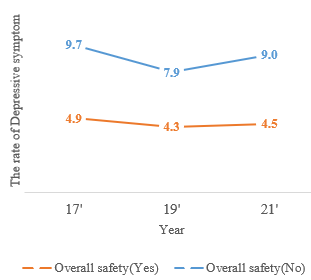 | 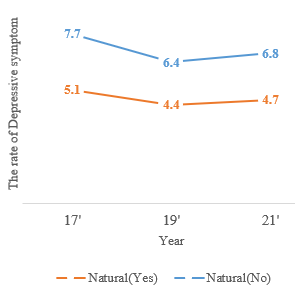 |
| --- | --- | --- | --- |
| 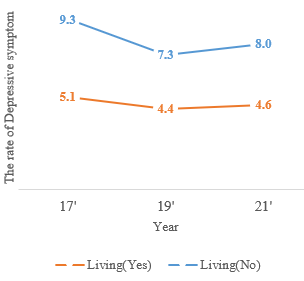 | 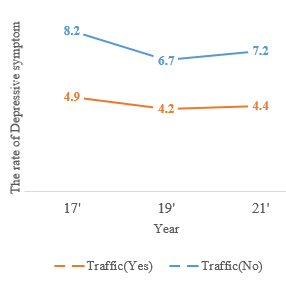 | 0.0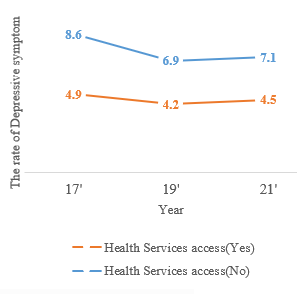 |  |

**Table S1. Questionnaire items used to measure indicators of perception of neighborhood**

Please indicate your thoughts on the neighborhood where you live.

| Categories | Indicators | Question | Answer |
| --- | --- | --- | --- |
| Social Network | - 1. Trust of neighbors | People in my neighborhood trust and rely on each other | 1.Yes, 2. No |
|  | - 1. Exchanging help with neighbors | People in my neighborhood are willing to help one another in times of need. | 1.Yes, 2. No |
|  | 2-1. Perceived satisfaction with overall safety | I am satisfied with the overall safety of my neighborhood (e.g., from natural disasters, traffic accidents, agricultural accidents, and crime). | 1.Yes, 2. No |
| Physical environment | 2-2. Perceived satisfaction with natural | I am satisfied with the natural environment of my neighborhood (e.g., parks, green spaces). | 1.Yes, 2. No |
|  | 2-3. Perceived satisfaction with living | I am satisfied with the living infrastructure in my neighborhood (e.g., electricity, water supply, garbage collection, sports facilities). | 1.Yes, 2. No |
|  | 2-4. Perceived satisfaction with traffic | I am satisfied with public transportation connections in my neighborhood (e.g., bus, taxi, subway, train). | 1.Yes, 2. No |
|  | 2-5. Perceived satisfaction with health services access | I am satisfied with the medical services available in my neighborhood (e.g., health centers, hospitals, Korean medicine clinics, pharmacies). | 1.Yes, 2. No |

**Table S2. P-values for Interaction Terms Between Time and Perception Indicators to Test the Parallel Trend Assumption (2017–2019)**

| Indicators | P-value for interaction terms  between Time(2017-2019) and Indicators |
| --- | --- |
| - 1. Trust of neighbors | 0.074 |
| - 1. Exchanging help with neighbors | 0.039 |
| 2-1. Perceived satisfaction with overall safety | 0.303 |
| 2-2. Perceived satisfaction with natural | 0.605 |
| 2-3. Perceived satisfaction with living | 0.274 |
| 2-4. Perceived satisfaction with traffic | 0.346 |
| 2-5. Perceived satisfaction with health services access | 0.293 |

**Table S3. Association between indicators of perception of neighborhood and depressive symptoms before and during the COVID-19 Pandemic according to occupation status**

Note: The model was adjusted for age group, sex, education, monthly income, marital status, the presence of hypertension or diabetes mellitus, and urbanity.

| Indicators | Occupation(Yes) | | DID | Occupation (No) | | DID |
| --- | --- | --- | --- | --- | --- | --- |
|  | Before the COVID-19 pandemic | During the COVID-19 pandemic | Wald χ2(P-value) | Before the COVID-19 pandemic | During the COVID-19 pandemic | Wald χ2(P-value) |
|  | aOR(95% CI) | aOR(95% CI) |  | aOR(95% CI) | aOR(95% CI) |  |
| Trust of neighbors (Yes) | 0.76(0.59, 0.97) | 0.43(0.35, 0.54) | 1.69(0.193) | 0.60(0.54, 0.67) | 0.49(0.44, 0.54) | 5.29(0.021) |
| Exchanging help with neighbors (Yes) | 0.77(0.62, 0.96) | 0.58(0.46, 0.72) | 1.21(0.271) | 0.72(0.64, 0.80) | 0.56(0.51, 0.62) | 3.06(0.080) |
| Perceived satisfaction with overall safety (Yes) | 0.64(0.48, 0.84) | 0.48(0.34, 0.68) | 0.32(0.572) | 0.52(0.45, 0.60) | 0.49(0.43, 0.56) | 2.11(0.147) |
| Perceived satisfaction with natural (Yes) | 0.65(0.51, 0.85) | 0.50(0.37, 0.67) | 0.08(0.778) | 0.63(0.55, 0.73) | 0.66(0.57, 0.76) | 0.16(0.692) |
| Perceived satisfaction with living (Yes) | 0.51(0.40, 0.65) | 0.53(0.40, 0.71) | 0.28(0.595) | 0.58(0.50, 0.68) | 0.54(0.47, 0.62) | 1.36(0.244) |
| Perceived satisfaction with traffic (Yes) | 0.78(0.63, 0.97) | 0.52(0.42, 0.64) | 4.42(0.036) | 0.61(0.54, 0.68) | 0.62(0.55, 0.69) | 1.69(0.194) |
| Perceived satisfaction with health services access (Yes) | 0.73(0.60, 0.90) | 0.51(0.41, 0.63) | 1.35(0.246) | 0.59(0.52, 0.66) | 0.64(0.57, 0.72) | 2.41(0.121) |

**Table S4. Association between indicators of perception of neighborhood and depressive symptoms before and during the COVID-19 Pandemic according to marital status**

Note: The model was adjusted for age group, sex, education, monthly income, occupation, the presence of hypertension or diabetes mellitus, and urbanity; “NA” presented “not available” due to lack of sample size.

| Indicators | Married (Live together) | | DID | Separated | | DID |
| --- | --- | --- | --- | --- | --- | --- |
|  | Before the COVID-19 pandemic | During the COVID-19 pandemic | Wald χ2(P-value) | Before the COVID-19 pandemic | During the COVID-19 pandemic | Wald χ2(P-value) |
|  | aOR(95% CI) | aOR(95% CI) |  | aOR(95% CI) | aOR(95% CI) |  |
| Trust of neighbors (Yes) | 0.64(0.55, 0.74) | 0.53(0.46, 0.61) | NA | 0.62(0.55, 0.70) | 0.46(0.40, 0.52) | 5.92(0.015) |
| Exchanging help with neighbors (Yes) | 0.76(0.66, 0.88) | 0.62(0.54, 0.72) | 0.95(0.329) | 0.72(0.63, 0.82) | 0.53(0.47, 0.60) | 4.00(0.045) |
| Perceived satisfaction with overall safety (Yes) | 0.55(0.46, 0.66) | 0.56(0.46, 0.69) | NA | 0.53(0.45, 0.62) | 0.46(0.39, 0.53) | 1.53(0.216) |
| Perceived satisfaction with natural (Yes) | 0.64(0.53, 0.76) | 0.65(0.53, 0.80) | 0.04(0.848) | 0.64(0.54, 0.75) | 0.62(0.53, 0.73) | 0.18(0.674) |
| Perceived satisfaction with living (Yes) | 0.56(0.47, 0.68) | 0.58(0.47, 0.72) | NA | 0.59(0.50, 0.69) | 0.52(0.44, 0.61) | 1.49(0.222) |
| Perceived satisfaction with traffic (Yes) | 0.63(0.54, 0.73) | 0.55(0.47, 0.63) | 0.01(0.936) | 0.63(0.56, 0.72) | 0.65(0.57, 0.74) | 0.25(0.616) |
| Perceived satisfaction with health services access (Yes) | 0.55(0.48, 0.64) | 0.61(0.53, 0.72) | NA | 0.65(0.57, 0.75) | 0.63(0.55, 0.72) | 0.00(0.970) |

**Table S5. Association between indicators of perception of neighborhood and depressive symptoms before and during the COVID-19 Pandemic according to the presence of hypertension or diabetes mellitus**

Note: The model was adjusted for age group, sex, education, monthly income, occupation, marital status, and urbanity; “NA” presented “not available” due to lack of sample size.

| Indicators | Underlying Disease (Yes) | | DID | Underlying Disease (No) | | DID |
| --- | --- | --- | --- | --- | --- | --- |
|  | Before the COVID-19 pandemic | During the COVID-19 pandemic | Wald χ2(P-value) | Before the COVID-19 pandemic | During the COVID-19 pandemic | Wald χ2(P-value) |
|  | aOR(95% CI) | aOR(95% CI) |  | aOR(95% CI) | aOR(95% CI) |  |
| Trust of neighbors (Yes) | 0.64(0.56, 0.73) | 0.48(0.43, 0.54) | 8.11(0.004) | 0.58(0.50, 0.67) | 0.49(0.42, 0.58) | 0.44(0.506) |
| Exchanging help with neighbors (Yes) | 0.74(0.66, 0.84) | 0.52(0.47, 0.59) | 7.58(0.006) | 0.71(0.61, 0.82) | 0.66(0.56, 0.77) | 0.02(0.876) |
| Perceived satisfaction with overall safety (Yes) | 0.49(0.42, 0.57) | 0.50(0.43, 0.59) | 2.19(0.139) | 0.65(0.53, 0.79) | 0.47(0.38, 0.57) | 0.46(0.496) |
| Perceived satisfaction with natural (Yes) | 0.59(0.51, 0.69) | 0.69(0.59, 0.82) | 0.10(0.747) | 0.73(0.60, 0.88) | 0.54(0.43, 0.66) | 1.71(0.191) |
| Perceived satisfaction with living (Yes) | 0.59(0.50, 0.70) | 0.57(0.49, 0.68) | 0.78(0.378) | 0.54(0.44, 0.66) | 0.47(0.38, 0.58) | 0.07(0.786) |
| Perceived satisfaction with traffic (Yes) | 0.59(0.52, 0.67) | 0.66(0.58, 0.74) | 0.97(0.326) | 0.72(0.61, 0.85) | 0.51(0.43, 0.59) | 0.86(0.354) |
| Perceived satisfaction with health services access (Yes) | 0.59(0.52, 0.67) | 0.63(0.56, 0.72) | 0.91(0.340) | 0.63(0.54, 0.74) | 0.61(0.51, 0.72) | 0.04(0.848) |
